# Extracellular annexin-A1 promotes myeloid/granulocytic differentiation of hematopoietic stem/progenitor cells via the Ca^2+^/MAPK signalling transduction pathway

**DOI:** 10.1038/s41420-019-0215-1

**Published:** 2019-09-23

**Authors:** Christiano M. V. Barbosa, Ricardo Ambrósio Fock, Araceli Aparecida Hastreiter, Cris Reutelingsperger, Mauro Perretti, Edgar J. Paredes-Gamero, Sandra H. P. Farsky

**Affiliations:** 1 Department of Clinical and Toxicological Analyses, School of Pharmaceutical Sciences, University of São Paulo, Sao Paulo, Brazil; 2 CARIM School for Cardiovascular Diseases, Maastricht University, 6200 MD Maastricht, The Netherlands; 3 Department of Biochemistry, Maastricht University, 6200 MD Maastricht, The Netherlands; 4 The William Harvey Research Institute, Bart’s and the London School of Medicine and Dentistry, Queen Mary University of London, London, UK; 5 Department of Biochemistry, Federal University of São Paulo, São Paulo, Brazil; 6 School of Pharmaceutical Sciences, Food and Nutrition (FACFAN), Federal University of Mato Grosso do Sul (UFMS), Campo Grande, MS 79070-900 Brazil

**Keywords:** Calcium signalling, Recombinant protein therapy

## Abstract

Annexin A1 (AnxA1) modulates neutrophil life span and bone marrow/blood cell trafficking thorough activation of formyl-peptide receptors (FPRs). Here, we investigated the effect of exogenous AnxA1 on haematopoiesis in the mouse. Treatment of C57BL/6 mice with recombinant AnxA1 (rAnxA1) reduced the granulocyte–macrophage progenitor (GMP) population in the bone marrow, enhanced the number of mature granulocytes Gr-1^+^Mac-1^+^ in the bone marrow as well as peripheral granulocytic neutrophils and increased expression of mitotic cyclin B1 on hematopoietic stem cells (HSCs)/progenitor cells (Lin^−^Sca-1^+^c-Kit^+^: LSK). These effects were abolished by simultaneous treatment with Boc-2, an FPR pan-antagonist. In in vitro studies, rAnxA1 reduced both HSC (LSKCD90^low^FLK-2^−^) and GMP populations while enhancing mature cells (Gr1^+^Mac1^+^). Moreover, rAnxA1 induced LSK cell proliferation (Ki67^+^), increasing the percentage of cells in the S/G2/M cell cycle phases and reducing Notch-1 expression. Simultaneous treatment with WRW4, a selective FPR2 antagonist, reversed the in vitro effects elicited by rAnxA1. Treatment of LSK cells with rAnxA1 led to phosphorylation of PCLγ2, PKC, RAS, MEK, and ERK1/2 with increased expression of NFAT2. In long-term bone marrow cultures, rAnxA1 did not alter the percentage of LSK cells but enhanced the Gr-1^+^Mac-1^+^ population; treatment with a PLC (U73122), but not with a PKC (GF109203), inhibitor reduced rAnxA1-induced phosphorylation of ERK1/2 and Elk1. Therefore, we identify here rAnxA1 as an inducer of HSC/progenitor cell differentiation, favouring differentiation of the myeloid/granulocytic lineage, via Ca^2+^/MAPK signalling transduction pathways.

## Introduction

Annexin A1 (AnxA1) is a member of the annexin superfamily, composed of 13 proteins. The protein binds to acidic phospholipids on the cell surface of activated cells and can also activate specific the G-protein-coupled receptors (GPCRs) termed formyl peptide receptors (FPR). AnxA1 displays high affinity for this target partners in the presence of Ca^2+^ (ref. ^1^). Secreted AnxA1 is a mediator of the anti-inflammatory activity of glucocorticoids, especially through regulation of neutrophil influx to the site of inflammation and stimulating resolution of the inflammatory process^2^. AnxA1 mediates a broad range of molecular and cellular processes, including intracellular vesicle trafficking^3^, tissue growth^4,5^, maintenance of the cytoskeleton and extracellular matrix integrity, kinase activities in signal transduction and differentiation^6^.

The effects of AnxA1 on blood cells seem to be specifically due to activation of FPR2 (refs. ^7,8^). By direct binding of AnxA1 on FPR2, heterotrimeric G proteins rapidly dissociate into α and βγ subunits. The βγ subunit initiates a series of signal transduction pathways, such as the one mediated by phospholipase Cγ (PLCγ)^9^, which could result in positive modulation of the mitogen-activated protein kinase (MAPK) pathway, particularly extracellular signal-related kinases 1 and 2 (ERK1/2)^10^. Another way to positively modulate MAPK signalling is through activation of Ca^2+^-sensing proteins, such as protein kinase C (PKC) and calmodulin^11,12^. Some of these mechanisms underlie the multiple biological properties of AnxA1 especially in the context of immune and hematopoietic settings. Herein, AnxA1 augments the activation of T cells with the involvement of the ERK and protein kinase B (AKT) pathways, favours their differentiation into Th1 cells^13^; affects the differentiation of semi-mature dendritic cells^14^; mediates the clearance of apoptotic neutrophils in the bone marrow^15^; inhibits neutrophil tissue accumulation by reducing leucocyte infiltration, activates neutrophil apoptosis^16^ and modulates secretion of stromal-derived factor-α (SDF-1α) by bone marrow stromal cells^17^.

In the bone marrow, haematopoiesis is initiated by a rare multipotent population called hematopoietic stem cells (HSCs), which, at each cell division, must decide whether to self-renew, differentiate, migrate, or die. HSCs can differentiate into common myeloid progenitors (CMPs), which then produce granulocytes and monocytes/macrophages. Endogenous chemical mediators control these processes by binding to specific cell-surface receptors in a stage-specific and lineage-specific manner, resulting in the activation of intracellular signal transduction pathways that are important for proliferation, survival, and differentiation^18,19^.

Definition of the mechanisms that regulate haematopoiesis is essential for successful mobilization of cells under stress conditions to defend the host^20^ and to rescue components of the blood during cancer chemotherapy or during haematological and immunosuppressive diseases^21,22^. Herein, we examined the role of exogenously administered AnxA1 in differentiation of murine HSCs/progenitor cells in the bone marrow, and our results identify this protein as an effective determinant to differentiate the myeloid/granulocytic lineage, through the engagement of the Ca^2+^/MAPK signalling transduction pathway.

## Results

### In vivo rAnxA1 treatment accelerates myeloid/granulocytic differentiation

**Fig. 1:**
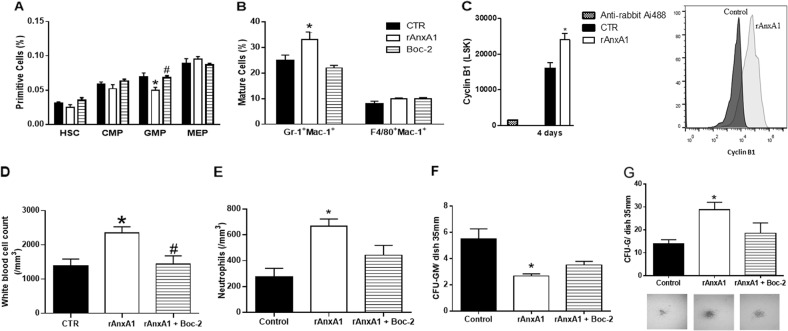
rANXA1 treatment promotes the granulocytic differentiation, neutrophilia, and enhances cyclin B1 expression in vivo: involvement of FPRs. Animals were treated with rANXA1 (1 mg/kg), once a day for 4 days. The pan-FPR antagonist Boc-2 (10 μg/mice) was carried out daily 1 h before rANXA1 treatment. **a** Percentage of primitive hematopoietic populations and **b** mature cells as quantified in the bone marrow of treated mice. **c** Expression of cyclin B on the LSK population. **d** Total white blood cell and the **e** neutrophil counts in the peripheral blood. **f** CFU-assay and the number of granulocyte–macrophage colony-forming cells (CFU-GM) and **g** granulocytic colony-forming cells (CFU-G) and representative pictures of colonies size (×4 magnification). Data express mean ± s.e.m. of five animals in each group. *P < 0.05 vs. control and ^#^*P* < 0.05 vs. rAnxA1. HSC hematopoietic stem cells, CMP, common myeloid progenitors, GMP, granulocyte–macrophage progenitors, MEP, megakaryocytic-erythroid progenitors, LSK Lin^−^Sca-1^+^c-Kit^+^

AnxA1 is an endogenous modulator of neutrophil trafficking between compartments^15,17^. To determine its effects on bone marrow cell maturation, mice were treated intraperitoneally with rAnxA1 once daily over 4 days, followed by bone marrow and peripheral blood collections. HSCs differentiate into hematopoietic multipotent progenitors (MPP), which is the first branch point of myeloid and lymphoid lineage. CMP are the first commitment myeloid lineage, which differentiate into granule-monocytic (GMP) or megakaryocytic-erythroid (MEP) progenitors. Treatment with the protein reduced the size of the GMP population (Fig. 1a) and increased the number of myeloid cells Gr-1^+^Mac-1^+^ in the bone marrow. These effects were not generalized to all differentiation lineages, as the monocytic population F4/80^+^Mac-1^+^Gr-1^−^ was not affected by the treatment (Fig. 1b). In addition, expression of mitotic cyclin B1 on HSCs/progenitor cells (LSKs) was augmented after treatment with rAnxA1 (Fig. 1c). rAnxA1 treatment was able to increase the number of white blood cells and neutrophils (Fig. 1d, e, respectively). Blockade of FPRs using the pan-antagonist Boc-2 reversed the (i) reduction in the number of GMP cells, (ii) increase in Gr-1^+^Mac-1^+^ population (Fig. 1a, b), and (iii) elevated number of white blood cells (Fig. 1d) due to AnxA1 treatment. The effect of Boc-2 on the elevated numbers of neutrophils detected after rAnxA1 treatment was not statistically significant (Fig. 1e).

Conversely, rAnxA1 treatment did not affect the proportions of other primitive cell populations such as HSCs, CMPs, and MEPs (Fig. 1a).

In view of these results, we performed additional experiments to evaluate the clonogenic capacity of bone marrow cells from rAnxA1-treated animals. To this end, colony-forming unit assays were performed, and the results indicated that rAnxA1 treatment reduced the number of granulocyte–monocyte colony-forming units (CFU-GM; Fig. 1f) while increasing the number of CFU-granulocytes (CFU-G) and size of the granulocytic colonies (Fig. 1g). Boc-2 treatment did not reverse the change in clonogenic capacity induced by rAnxA1 (Fig. 1f, g).

**Fig. 2:**
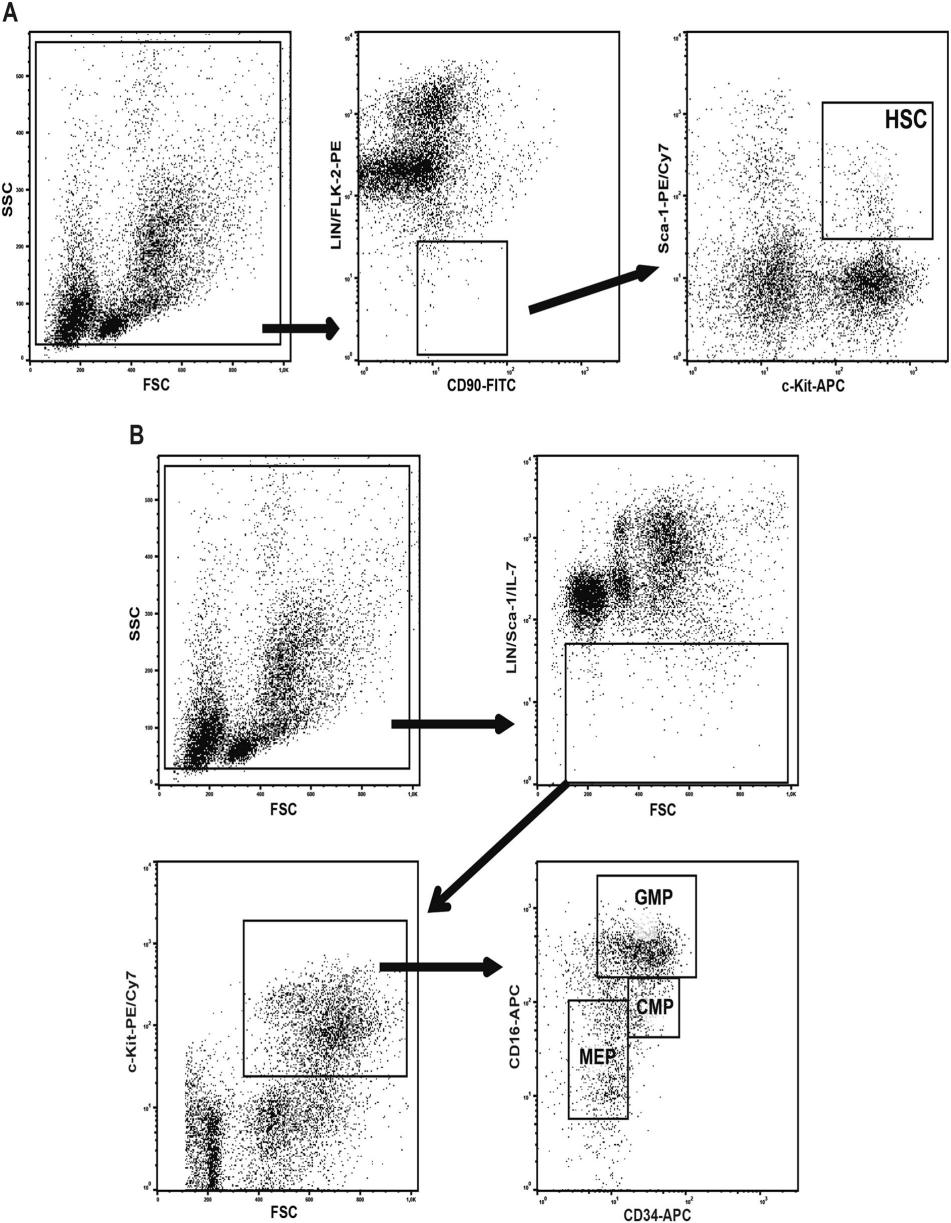
The regions analysed for each murine hematopoietic population are shown. **a** Hematopoietic stem cells (HSC), **b** common myeloid progenitors (CMP), granulocytic–monocytic progenitors (GMP), and megakaryocytic-erythroid progenitors (MEP)

The gating and staining strategy used to identify cell populations is provided in Fig. 2.

### rAnxA1 modifies the proliferation and differentiation of bone marrow hematopoietic cells

**Fig. 3:**
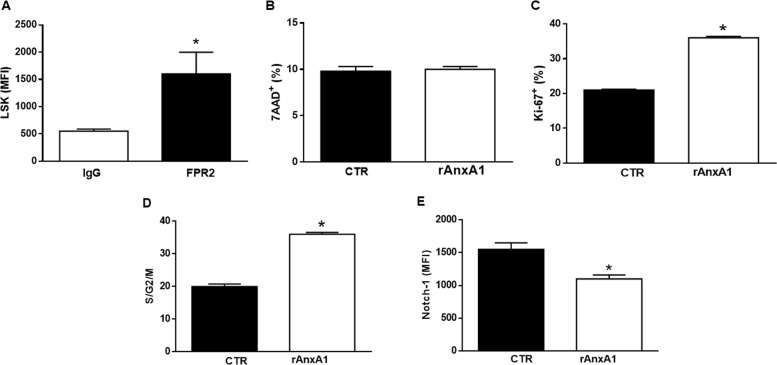
rAnxA1 increases proliferation and decreases Notch-1 expression. Bone marrow cells were collected from naïve mice and stimulated with rAnxA1 (100 nM) in for 12 h. **a** FPR2 expression in LSK cells. Analysis in LSK cells: **b** viability; **c** expression of Ki67; **d** percentage of cells in S/G2/M cycle phases; **e** expression of undifferentiated marker Notch-1. Data express mean ± s.e.m. of cells collected from five animals in each group. *P < 0.05 vs. control. LSK Lin^−^Sca-1^+^c-Kit^+^

Based on these in vivo properties of rAnxA1, in vitro experiments were carried out to investigate the mechanisms of action of the recombinant protein on myeloid cells. We first determined the positive expression of FPR2 in HSCs/progenitor cells (Fig. 3a). Afterwards, bone marrow cells were stimulated in vitro with rAnxA1 (12 h at 100 nM) to investigate the effects on HSCs/progenitor cells (LSK). This treatment did not affect cell viability, determined by 7-AAD (Fig. 3b) or annexin V (Fig. 1S) staining. Additionally, stimulation of bone marrow cells with rAnxA1 increased proliferation of LSK cells, as indicated by (i) the augmented percentage of high proliferative Ki67-positive cells (Fig. 3c) as well as (ii) the increased percentage of LSK cells in the S/G2/M cell cycle phases (Fig. 3d). These results identify rAnxA1 as an inducer of proliferation of primitive hematopoietic cells.

To verify the ability of rAnxA1 to promote myeloid/granulocytic differentiation, we quantified Notch receptor expression. The data obtained showed reduced expression of Notch-1 on LSK cells after addition of rAnxA1 in vitro (Fig. 3e). Of note, the Notch receptor, an essential regulator of hematopoietic differentiation, is expressed in undifferentiated progenitors of bone marrow cells^23,24^.

**Fig. 4:**
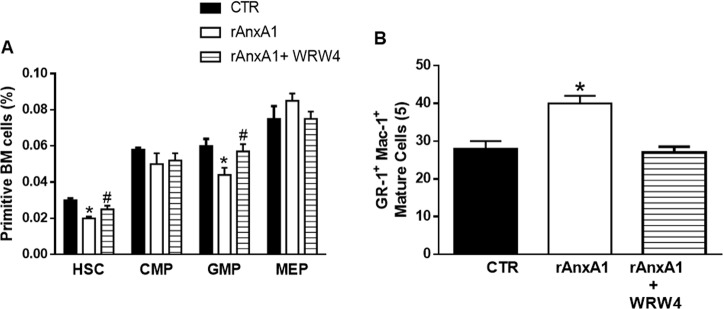
rAnxA1 decreases the proportion of differentiated primitive hematopoietic cell populations via FPR2. Bone marrow cells were stimulated with rAnxA1 (100 nM) for 12 h. WRW_4_ (10 μM) was applied to cells 1 h before rAnxA1. Cells were analysed by flow cytometry. **a** Percentages of primitive cells and **b** mature cells in the bone marrow. Data express mean ± s.e.m. of cells collected from five animals in each group. *P < 0.05 vs. control and ^#^*P* < 0.05 vs. rAnxA1. HSC hematopoietic stem cells, CMP common myeloid progenitors, GMP granulocyte–macrophage progenitors, MEP megakaryocytic-erythroid progenitors

Next, we evaluated the percentages of the primitive and mature hematopoietic populations, including HSCs, CMPs, GMPs, and MEPs from bone marrow cells stimulated in vitro with rAnxA1 (12 h at 100 nM). rAnxA1 incubation decreased the proportion of HSC and GMP in short-term culture (Fig. 4a), paralleled by a significant increase in the mature granulocytic (Gr-1^+^Mac-1^+^) population (Fig. 4b). The CMP and MEP populations were not affected (Fig. 4a).

Treatment with WRW_4_, a selective FPR2 antagonist, abolished the decrease in primitive hematopoietic cell populations (HSC and GMP) induced by rAnxA1 (Fig. 4a) as well as the effect of rAnxA1 on mature granulocytic (Gr-1^+^Mac-1^+^) cells (Fig. 4b).

### rAnxA1 activates intracellular PLCγ2, PKC, and Ras/MEK/ERK pathways and NFAT2 expression in BM LSK cells

**Fig. 5:**
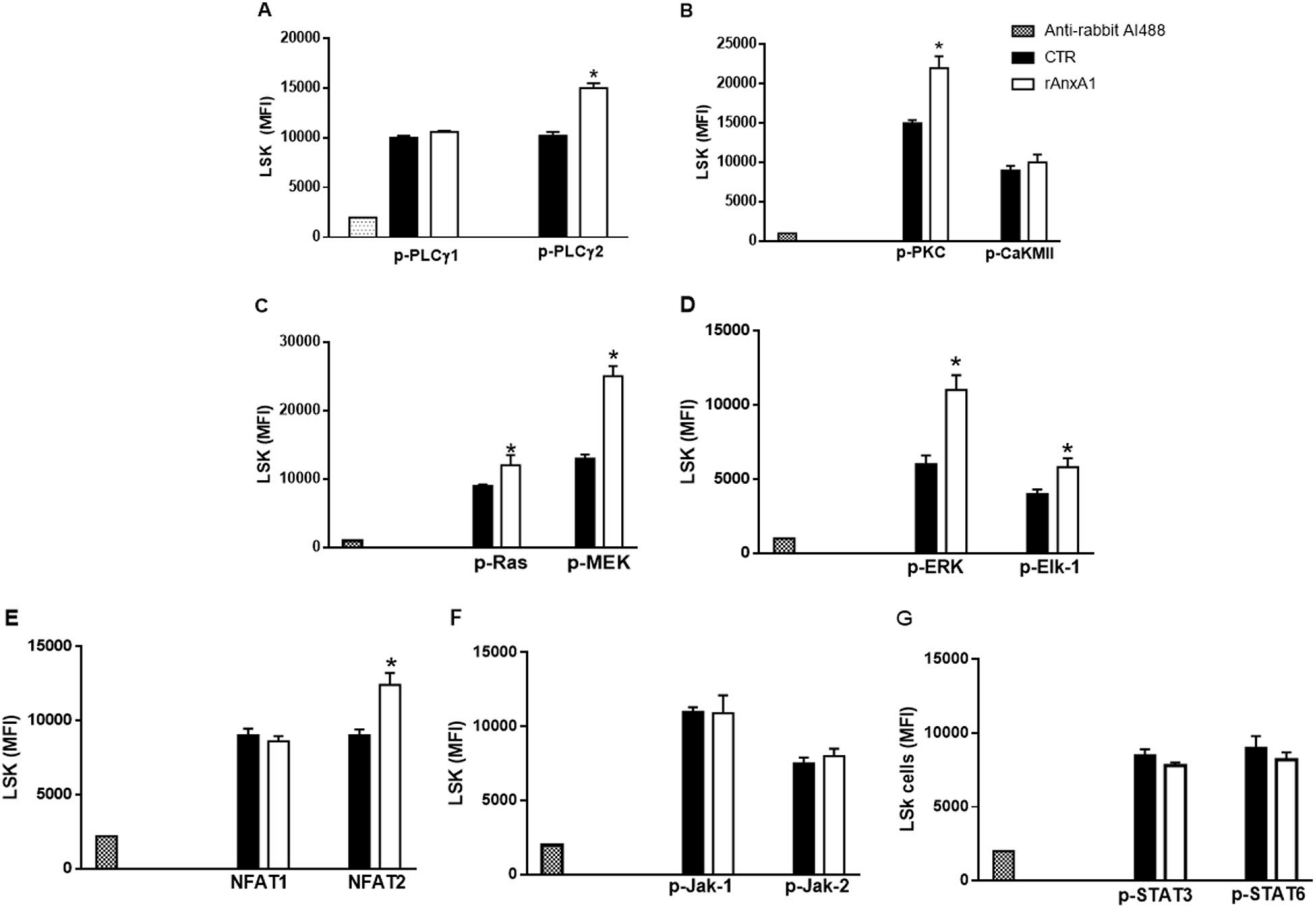
rAnxA1 activates PLCγ2, PKC, Ras/MEK/ERK and increases the expression of NFAT2. Bone marrow cells were stimulated with rAnxA1 (100 nM) for 10 min to measure p-PLCγ1, p-PLCγ2, p-PKC, p-CaMKII, p-Ras, p-MEK, p-Jak-1, p-Jak-2, p-STAT-3, and p-STAT-5 expressions or 30 min to monitor p-ERK, p-Elk-1, NFAT1, and NFAT2 levels. Data were collected in LSK cells by flow cytometry using antibodies against **a** p-PLCγ1 and p-PLCγ2; **b** p-PKC and p-CaMKII; **c** p-Ras and p-MEK; **d** p-ERK and p-Elk-1; **e** NFAT1 and NFAT2; **f** p-Jak-1 and p-Jak-2; **g** p-STAT-3 and p-STAT-5. Data express mean ± s.e.m. of cells collected from three animals in each group. *P < 0.05 vs. control

Our results suggest that rAnxA1 mediates myeloid/granulocytic differentiation, reducing the number of primitive hematopoietic cells and increasing the relative proportion of cells in their mature forms, an effect obtained through the GPCR FPR2. Therefore, next we investigated some of the downstream signalling pathways potentially modulated in these settings and involved in myeloid differentiation. Bone marrow cells were stimulated by rAnxA1 (100 nM; 10 or 30 min), and phosphorylation of specific signalling elements was quantified by flow cytometry, starting from Ca^2+^-sensing proteins related to myeloid differentiation^25,26^. We observed increased phosphorylation of the PLCγ2 isoform and PKC after stimulation with rAnxA1 (Fig. 5a, b); in contrast, CaMKII was not activated by rAnxA1 (Fig. 5b).

On the same vein, rAnxA1 treatment evoked phosphorylation of Ras, MEK, ERK1/2, and ELK proteins (Fig. 5c, d); activation of the calcineurin-NFAT signalling, with increased expression of NFAT2 but not NFAT1 (Fig. 5e). Treatment of cells with rAnxA1 did not activate either isoform of JAK1/JAK2 (Fig. 5f) or STAT3/STAT5 (Fig. 5g). Specific histograms depicting the flow cytometry results are shown in Figure S2.

### rAnxA1 induces granulocytic differentiation in long-term bone marrow cultures

**Fig. 6:**
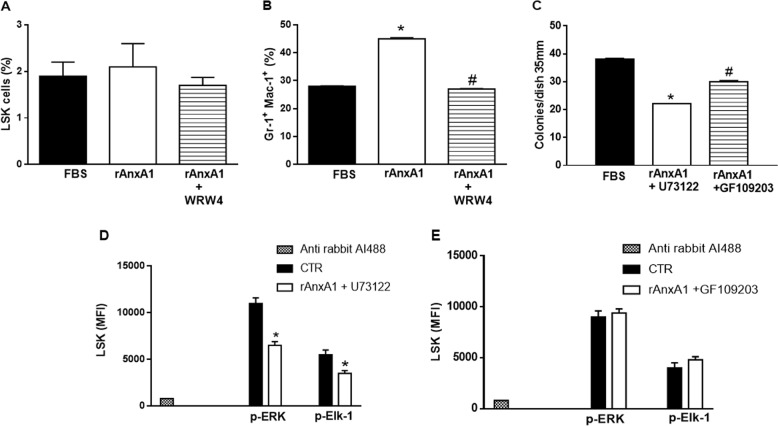
rAnxA1 increases mature myeloid cells in long-term bone marrow cultures (LTBMC) and pharmacological inhibitors of PLC and PKC affects its clonogenic capability. LTBMC were established (see Methods) and incubated with rAnxA1 (100 nM) with or without 10 µM WRW_4_. Flow cytometry was then performed to quantify: **a** percentage of LSK cells; **b** mature Gr-1^+^Mac-1^+^ population. Moreover, **c** CFU assays was performed in presence or absence of U73122 (PLC inhibitor) or GF109203 (PKC inhibitor). **d** Expression of p-ERK and p-Elk-1 in LSK cells treated with PLC inhibitor or **e** PKC inhibitor. Data express mean ± s.e.m. of cells collected from five animals in each group. *P < 0.05 vs. control and ^#^*P* < 0.05 vs. rAnxA1

To evaluate granulocytic differentiation, long-term bone marrow culture (LTMBC) was employed following Dexter’s (1977)^27^ protocol as a way to sustain HSC and myeloid populations in vitro. The percentage of the LSK population was not affected by rAnxA1 treatment (Fig. 6a). However, the Gr-1^+^Mac-1^+^ population increased in response to rAnxA1 (Fig. 6b). The effects were mediated by FPR2, as pre-treatment with WRW_4_ abolished these responses evoked by addition of rAnxA1 (Fig. 6b).

### Inhibitor of PLC, MEK, or PKC blocks colony formation evoked by rAnxA1

After evaluating the role of exogenous AnxA1 in the differentiation of hematopoietic precursors in in vivo and in vitro systems, we queried a potential cross-talk between Ca^2+^ signalling and the MEK/ERK pathway using pharmacological inhibitors. U73122, a PLC inhibitor, reduced the number of colonies formed by rAnxA1 (Fig. 6c) and at the same time reduced the extent of ERK1/2 and Elk-1 phosphorylation (Fig. 6d). GF109203, a PKC inhibitor, also reduced the number of colonies formed (Fig. 6c) without affecting phosphorylation of ERK1/2 and Elk-1 (Fig. 6e) upon rAnxA1 application.

These results demonstrated the interactions between classical intracellular Ca^2+^-dependent kinases and pathways regulating cell proliferation and differentiation, such as MAPK pathways. All histograms of flow cytometry are shown in Fig. S2.

## Discussion

Neutrophils are versatile cells, able to exert critical immunoregulatory roles beyond those involved in the first line of defense during innate immunity. In the emergency granulopoiesis that maintains sufficient peripheral neutrophil numbers under stress conditions, neutrophils migrate to draining lymph nodes to regulate T-cell^28–31^ and B-cell activation, plasma cell generation, and antibody production^32–34^. In the spleen, neutrophils support B-cell functions in antibody production^35,36^; can reverse transendothelial migration into sites of inflammation and return to the circulation to propagate inflammation^37,38^ and play a pivotal role in the resolution of inflammation^39,40^. Therefore, emergency granulopoiesis must occur promptly and transiently to rescue homoeostasis.

We previously demonstrated that endogenous AnxA1, secreted by differentiating granulocytes in the bone marrow and blood neutrophils, controls the trafficking of neutrophils across compartments within the body^15,17^. Herein, we identify rAnxA1 as an inducer of differentiation of the myeloid/granulocytic lineage, through involvement of the Ca^2+^/MAPK signalling transduction pathway, which suggests that this mediator can be effective as an adjuvant tool to rescue granulopoiesis.

The role of rAnxA1 in in vivo granulopoiesis was first identified by quantifying the percentage of HSC and myeloid precursor cells. HSCs self-renew and differentiate into all the cells of the hematopoietic system, and they are responsible for lifelong blood production. Under normal conditions, HSCs are found in bone marrow in specialized niche microenvironments that are critical for their maintenance and functional activity^41^. HSCs differentiate into MPP, hierarchically giving rise to lineage-specific progenitors. The first myeloid lineage is recognized as CMP, which can differentiate into GMP or MEP progenitors, giving rise to all myeloid mature cells^42^. rAnxA1-treated mice presented reduced percentages of the GMP while increasing the proportion of the mature granulocytic population in the bone marrow, actions which coincided with a higher number of neutrophils in the blood. Congruently, higher numbers of granulocytic colonies were formed when bone marrow collected from rAnxA1 mice was cultivated in methylcellulose. These data, along with increased expression of cyclin B1 in HSC/progenitor cells, pointed out rAnxA1 as an inducer of granulocytosis.

The functional involvement of AnxA1 targets like the FPRs was evinced through the use of antagonists against these receptors. Of interest, recent work has shown that activation of FPR2 by another agonist like the synthetic peptides WKYMV induces granulopoesis^43^. Using pharmacological approaches, we show that AnxA1 specifically induces granulopoiesis by activating FPR2, whereas the monocytic population F4/80^+^Mac-1^+^Gr-1^−^ was not affected by rAnxA1 treatment. In vitro rAnxA1 treatment corroborated the in vivo actions of rAnxA1 and revealed at least some of the intracellular pathways activated by the protein. rAnxA1 induced LSK cell proliferation, showing the ability of rAnxA1 to reduce the number of primitive hematopoietic cells in the quiescent state. In the mean time, reduced expression of Notch-1 by LSK cells corroborated the action rAnxA1, as lower levels of Notch-1 have been associated with the self-renewal, quiescence, maintenance, and differentiation of HSCs^44,45^. Additional data obtained in LTBMCs confirmed the ability of rAnxA1 to increase the Gr-1^+^Mac-1^+^ population. Together, the data obtained confirmed rAnxA1 as an inducer of myeloid/granulocytic differentiation.

Binding of AnxA1 to FPR2 activates intracellular pathways in neutrophils mainly related to inhibition of inflammation, such as those which halt the process and elicit neutrophil apoptosis and their efferocytosis by macrophages^16,46–48^. The downstream intracellular pathways triggered by AnxA1 in granulopoiesis are unknown. Here we show a functional involvement of Ca^2+^_i_-dependent kinases and the ERK1/2 pathway in rANXA1 regulation of murine HSCs/progenitor myeloid cell differentiation. Incubation of LSK cells with rAnxA1 increased the phosphorylation of proteins such as Ras, MEK, and ERK1/2. Moreover, rAnxA1 increased the activation of the transcription factors Elk-1 and NFAT2. Indeed, Ca^2+^-dependent proteins, such as PLCγ, PKC, and CaMKII, are present in HSC/progenitor cells, and their absence results in hematopoietic failure, characterized by a diminished LSK cell population^49^. Therefore, inhibition of Ca^2+^-kinase-dependent substrates can modulate MEK/ERK activation by acting as key intermediate modulators in the cascade of granulocyte differentiation. In the same set of experiments, we also pointed out that the activation of ERK1/2 by rAnxA1 is dependent on PLC, as treatment with the pharmacological inhibitor of PLC (U73122) reduced AnxA1-induced ERK1/2 activation in murine hematopoietic HSC/progenitor cells. Treatment with GF109203, a pharmacological inhibitor of PKC, did not impact on ERK1/2 activation. However, as inhibitors of PLC or PKC reduced the number of colonies produced by rAnxa1, there is involvement of at least a distinct pathway in these actions. In line we our data, activation of PLC has been reported in granulopoiesis evoked by an FPR2 agonist^43^.

**Fig. 7:**
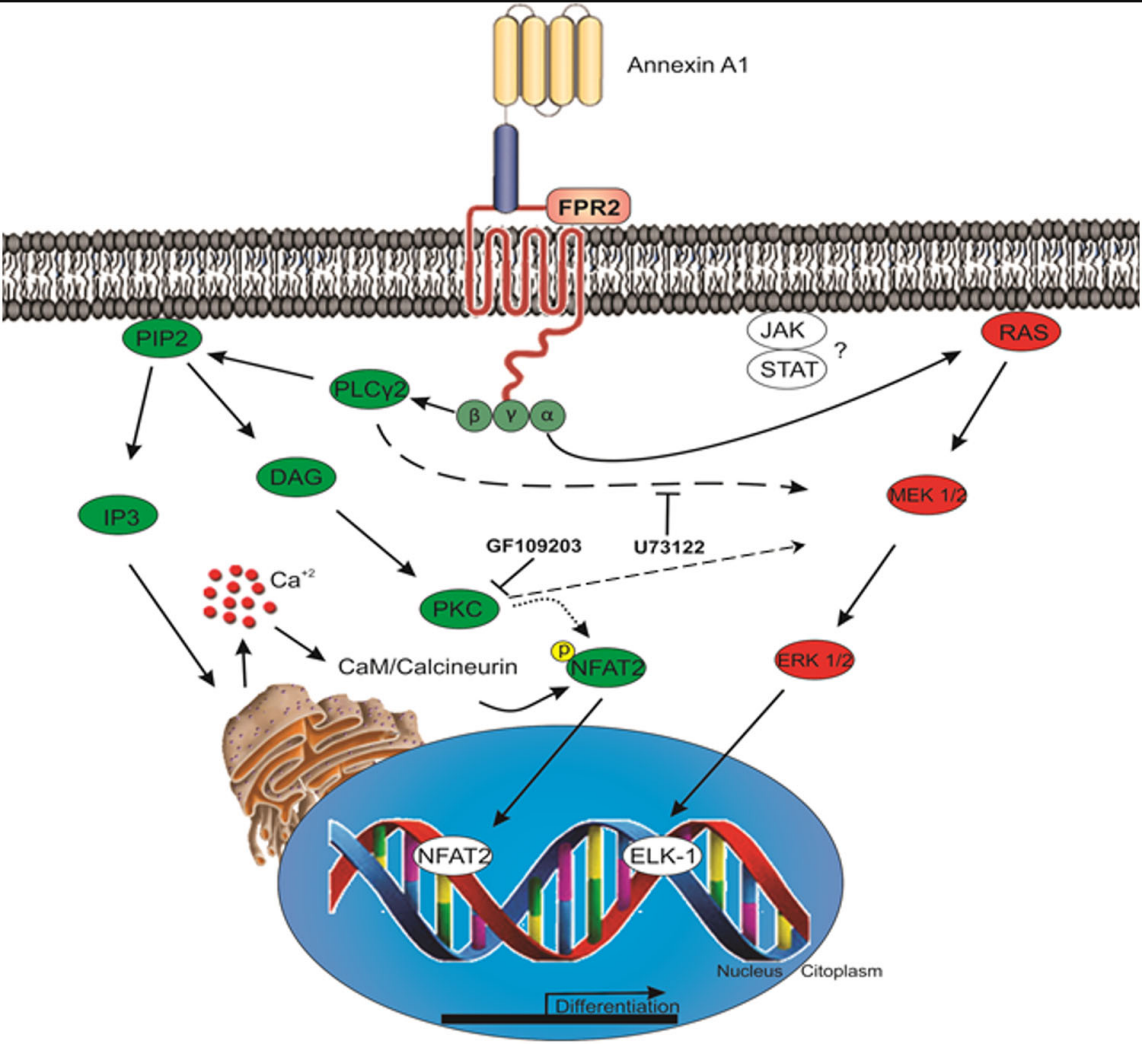
Schematic for the proposed mechanism underlying the effects of rAnxA1 on myeloid/granulocytic differentiation. Annexin A1 binds to membrane FRP2, a G-protein coupled receptor, and activates G protein. Activated β subunit down streams phospholipase Cγ2 (PLCγ2), phosphatidylinositol 3,4-bisphosphate (PIP2), inositol triphosphate (IP3), and diacylglycerol (DAG). IP3 mobilizes intracellular pool of calcium, which activates calmodulin/calcineurin (CaM/Calcineurin). DAG also activates protein kinase C (PKC). PKC and CaM/Calcineurin activates nuclear factor activated of T cells 2 (NFAT2) to induce differentiation. α subunit downstream RAS, MEK1/2, ERK1/2. ERK1/2 activates ELK-1 and cell differentiation

Altogether, we report, for the first time that rAnxA1 activates myeloid/granulocytic differentiation via FPR2 activation. These effects are married by a decrease in the HSC and GMP populations, and increased production of granulocytes. We propose a duality in signalling triggered by AnxA1 in primitive hematopoietic cells, with activation of both the MEK/ERK1/2 and Ca^2+^-dependent kinases PLCγ2/PKC pathway. Elk-1 and NFAT2 transcription factors were also associated with the effects elicited by AnxA1 (Fig. 7). Therefore, these new data created the scientific background onto which develop AnxA1 as a novel therapeutic tool to induce granulocyte differentiation in cases of emergency granulopoiesis.

## Materials and methods

### Animals

Male 8- to 12-week-old C57BL/6 mice were acquired from the Animal Housing Facilities of the Federal University of São Paulo (São Paulo, Brazil). All experiments were approved by the Animal Care and Ethics Committee of the School of Pharmaceutical Sciences, University of São Paulo (CEUA/FCF 31.2016-P519).

### In vivo treatments

#### Pharmacological administrations

Animals were treated once a day with rAnxA1 (provided by Dr. Chris Reutelingsperger; 1 mg/kg; i.p.), for four consecutive days. Another set of animals was treated simultaneously with Boc-2 (MP Biomedicals, USA; 10 μg/mice, i.p.) a pan-antagonist to FPRs. This protocol was based on the one described by Machado et al. (2016)^17^. Control mice received sterile saline solution by the same route and schedule of treatments. Four hours after the last injection, animals were anaesthetized with xylazine chlorohydrate (Rompum^®^, Bayer, Brazil; 10 mg/kg) and ketamide chlorohydrate (Ketamina^®^, Cristália, Brazil; 100 mg/kg) and euthanized.

#### Immunophenotyping of bone marrow cells

Bone marrow cells were obtained from the different groups of mice by flushing the femoral cavities with 2 mL of Dulbecco’s modified Eagle’s medium (DMEM) supplemented with 0.5% foetal bovine serum (Atená, Brazil); the percentage of primitive hematopoietic cell populations was quantified by flow cytometry. The percentages of each hematopoietic population were determined by flow cytometry as described by Barbosa et al. (2011)^50^. Several antibodies were used to recognize subsets rich in HSCs (CD90^low^Lin^−^FLK-2^−^Sca-1^+^c-Kit^+^), CMPs (Lin^−^Sca-1^−^IL7R^−^c-Kit^+^CD34^+^CD16^low^), GMPs (Lin^−^Sca-1^−^IL7R^−^c-Kit^+^CD34^+^CD16^high^), MEPs (Lin^−^Sca-1^−^IL7R^−^c-Kit^+^CD34^−^CD16^low^), and LSK cells (Lin^−^Sca-1^+^c-Kit^+^). The mature myeloid populations (F4/80-Alexa Fluor 488, Gr-1-PE and CD11b-Cy7/PE) were evaluated with antibodies that recognize the granulocytic population (Gr-1^+^CD11b^+^F4/80). A total of 2 × 10^6^ cells were used to identify and quantify bone marrow populations. All these sets of antibodies were purchased from Becton Dickinson (USA) or eBioscience (USA).

HSCs were defined in this study as LSK CD90^low^FLK-2^−^ cells. Cyclin B1 expression was quantified in LSK population; the cells were collected, fixed in 2% paraformaldehyde solution for 30 min, and permeabilized for 15 min with 0.001% Triton X-100 to be incubated with anti-cyclin B1 antibody (Cell Signaling Technologies, USA) for 2 h, then with the secondary antibody rabbit Anti-IgG Alexa Fluor 488 (Molecular Probes/Invitrogen, USA) for 40 min. Analyses were performed using an Accuri C6 flow cytometer (Becton Dickinson, USA) and FlowJo software. The gating strategy used to evaluate the primitive murine hematopoietic populations is shown in Fig. 2.

#### White blood cell count

Blood samples were collected in tubes with EDTA as an anticoagulant (Sigma Chemical Co., USA), and a blood cell count was performed on ABX Micros ABC Vet equipment (Horiba ABX, France). Morphological and leucocyte differentiation analyses were performed on blood smears stained with standard May–Grünwald–Giemsa solutions (Sigma Chemical Co., USA).

### In vitro treatments

#### Viability and cell cycle

Bone marrow cells from untreated animals were collected as described above. First, we measured the expression of FPR2 in LSK cells. The anti-FPR2-FITC antibody was used (Bioss, Massachusetts, USA) to quantify this expression by flow cytometry. Afterwards, bone marrow cells were treated with rAnxA1 (100 nM) for 12 h, and viability of the LSK population determined using 7-aminoactinomycinD (7-AAD; Sigma Aldrich, USA); cell cycle phases and expression of Ki67 and Notch-1 were also evaluated. These quantifications were carried out by flow cytometry. Cells were fixed in 2% paraformaldehyde for 30 min, washed with 0.1 M glycine, and permeabilized with 0.001% Triton X-100. Subsequently, 2 × 10^6^ cells were incubated with primary anti-cyclin B1, anti-Notch-1 or anti-Ki67 (Cell Signaling Technology) antibodies for 2 h, and then 40 min with secondary rabbit Anti-IgG Alexa Fluor 488 (Molecular Probes/Invitrogen, USA). Analyses were performed on the LSK population gated as described above, using an Accuri C6 flow cytometer (Becton Dickinson, USA) and the FlowJo software.

#### Methylcellulose colony-forming unit (CFU) assay

To analyse the colony-forming units derived from HSC, bone marrow cells from animals treated as described above were collected, and mononuclear cells were separated by density gradient centrifugation using the Ficoll-Histopaque protocol (Sigma Aldrich, USA and 5 × 10^4^ cells/mL were platted in MethoCult™ media (GF M3534, STEMCELL Technologies, Inc., Canada). The mixture was allowed to stand for 3 min to remove bubbles, and 1 ml was dispensed by a pipette into each well of a six-wells cell culture dish (in duplicate). Cells were incubated for 14 days in a 5% CO_2_ incubator at 37 °C. After 14 days of culture, total colonies were counted under an optical microscope at 40× magnification (Carl-Zeiss, Germany), and the numbers of granulocyte–macrophage colony-forming cells (CFU-GM) and granulocytic colony-forming cells (CFU-G) were quantified.

#### Bone marrow cell culture

Bone marrow cells, obtained as described above, were incubated in 12-well plates (10^6^ cells per well) in the presence or absence of WRW4 (10 μM; Tocris Bioscience, UK), a selective antagonist of FPR2^51,52^, for 1 h. Then, cells were treated with rAnxA1 (100 nM) for 12 h at 37 °C in 5% CO_2_ and immunophenotyped as above.

#### Protein expression and phosphorylation status

Bone marrow cells from untreated animals were collected. Cells were treated in vitro with rAnxA1 (100 nM) for 10–30 min depending on specific signalling readouts and fixed in 2% paraformaldehyde for 30 min, washed with 0.1 M glycine, and permeabilized with 0.01% saponin. Subsequently, 2 × 10^6^ cells were incubated with anti-p-PLCγ1_Tyr783_, p-PLCγ2_Tyr759_, p-CaMKII_thr286_, p-PKC_Thr514_, p-JAK-1_Tyr1022/1023_, p-JAK-2_Tyr1008_, p-Ras, P-MEK, p-ERK1/2_Thr202/Thr204_, p-STAT3_Tyr705_, p-STAT5_Tyr694_, p-Elk-1, NFAT1, or NFAT2 primary antibodies for 2 h at 4 °C. All primary antibodies were purchased from Cell Signaling Technology (USA). Secondary labelling was carried out by incubation with Alexa Flour 488-conjugated rabbit Anti-IgG (Molecular Probes/Invitrogen, USA) for 40 min. Analyses were performed on the LSK population gated as described above, using an Accuri C6 flow cytometer (Becton Dickinson, USA) and the FlowJo software.

#### Long-term bone marrow cultures

Long-term bone marrow cultures (LTBMCs) were prepared as previously described by Dexter et al. ^27^ with some modifications. Briefly, 10^6^ total bone marrow cells were plated into 12-well plates and fed weekly with Iscove’s modified Dulbecco’s medium (IMDM) supplemented with 12.5% foetal bovine serum, 12.5% horse serum (StemCell Technologies, Canada) and 1 μM hydrocortisone (Sigma Aldrich, USA). Cultures were maintained at 37 °C under 5% CO_2_. After establishment of a confluent stromal layer, remaining non-adherent cells were removed, and 10^6^ bone marrow mononuclear cells per well were added to the pre-cultured stroma, along with fresh media. After 1 week of co-culture, cells were further cultured in IMDM with 0.5% FBS for 24 h. Subsequently, LTMBCs were pre-incubated with or without WRW4 (10 µM) for 1 h and then treated with rAnxA1 (100 nM). After 3 days, cells were collected for immunophenotyping using antibodies and protocols described.

#### Cell signalling assays using pharmacological inhibitors

CFU assays were performed as described previously. Murine bone marrow cells (5 × 10^4^) were plated in methylcellulose-based medium (Methocult M3534; Stem Cell Technologies, USA) with AnxA1 (100 nM) and signalling inhibitors of PLC (U73122; 5 µM/) or PKC (GF109203; 10 nM)^53^, and total number of colonies counted after 3 days under an optical microscope at ×40 magnification (Carl-Zeiss, Germany). An additional set of experiments was also performed: here bone marrow cells were collected, treated with the inhibitors of PLC or PKC for 1 h, and subsequently stimulated with 100 nM AnxA1 for 30 min. Expression of p-ERK and p-Elk-1 was quantified by flow cytometry as above.

### Statistical analyses

The data sets were subjected to normality tests and analysed by Student’s *t*-test or one- or two-way ANOVA followed by Bonferroni’s post hoc test. The level of significance adopted was 95% (*p* < 0.05). All data are represented as mean ± standard error of the mean (SEM). The number of mice analysed in each experiment is denoted by *n*, as detailed in specific figure legends. Statistical analyses were performed using GraphPad Prism^®^ 5 (GraphPad Software Inc., La Jolla, USA).

## Supplementary information

Suppl. Figure 2

Suppl. Figure 1

Supplemental Material File #1

## References

[1] Perretti M, D’Acquisto F (2009). Annexin A1 and glucocorticoids as effectors of the resolution of inflammation. Nat. Rev. Immunol..

[2] Headland SE, Norling LV (2015). The resolution of inflammation: principles and challenges. Semin. Immunol..

[3] Gerke V, Creutz CE, Moss SE (2005). Annexins: linking Ca^2+^ signalling to membrane dynamics. Nat. Rev. Mol. Cell. Biol..

[4] Petrella A (2005). Induction of annexin-1 during TRAIL-induced apoptosis in thyroid carcinoma cells. Cell Death Differ..

[5] Scannell M, Maderna P (2006). Lipoxins and annexin-1: resolution of inflammation and regulation of phagocytosis of apoptotic cells. Sci. World J..

[6] Gerke V, Moss SE (2002). Annexins: from structure to function. Physiol. Rev..

[7] Gavins FN, Kamal AM, D’Amico M, Oliani SM, Perretti M (2005). Formyl-peptide receptor is not involved in the protection afforded by annexin 1 in murine acute myocardial infarct. FASEB J..

[8] Hayhoe RP (2006). Annexin 1 and its bioactive peptide inhibit neutrophil-endothelium interactions under flow: indication of distinct receptor involvement. Blood.

[9] Ferry X, Eichwald V, Daeffler L, Landry Y (2001). Activation of betagamma subunits of G(i2) and G(i3) proteins by basic secretagogues induces exocytosis through phospholipase C beta and arachidonate release through phospholipase C gamma in mast cells. J. Immunol..

[10] Buckley CT, Sekiya F, Kim YJ, Rhee SG, Caldwell KK (2004). Identification of phospholipase c-gamma 1 as a mitogen-activated protein kinase substrate. J. Biol. Chem..

[11] Migeotte I, Communi D, Parmentier M (2006). Formyl peptide receptors: a promiscuous subfamily of G protein-coupled receptors controlling immune responses. Cytokine Growth Factor Rev..

[12] Bena S, Brancaleone V, Wang JM, Perretti M, Flower RJ (2012). Annexin A1 interaction with the FPR2/ALX receptor: identification of distinct domains and downstream associated signaling. J. Biol. Chem..

[13] D’Acquisto F (2007). Impaired T cell activation and increased Th2 lineage commitment in annexin-1-deficient T cells. Eur. J. Immunol..

[14] Huggins A, Paschalidis N, Flower RJ, Perretti M, D’Acquisto F (2009). Annexin-1-deficient dendritic cells acquire a mature phenotype during differentiation. FASEB J..

[15] Dalli J (2012). Annexin A1 regulates neutrophil clearance by macrophages in the mouse bone marrow. FASEB J..

[16] Sugimoto, M. A., Vago, J. P., Teixeira, M. M., Sousa, L. P. Annexin A1 and the resolution of inflammation: modulation of neutrophil recruitment, apoptosis, and clearance. *J. Immunol. Res*. 8239258 (2016). 10.1155/2016/8239258 PMC4738713 26885535

[17] Machado ID (2016). Annexin A1 is a physiological modulator of neutrophil maturation and recirculation acting on the CXCR4/CXCL12 pathway. J. Cell Physiol..

[18] Weissman IL (2000). Translating stem and progenitor cell biology to the clinic: barriers and opportunities. Science.

[19] Miranda MB, Johnson DE (2007). Signal transduction pathways that contribute to myeloid differentiation. Leukemia.

[20] Nauseef WM, Borregaard N (2014). Neutrophils at work. Nat. Immunol..

[21] Bendall LJ, Bradstock KF (2014). G-CSF: from granulopoietic stimulant to bone marrow stem cell mobilizing agent. Cytokine Growth Factor Rev..

[22] Takeshima T (2016). Key role for neutrophils in radiation-induced antitumor immune responses: potentiation with G-CSF. Proc. Natl. Acad. Sci. USA.

[23] Milner LA, Kopan R, Martin DI, Bernstein ID (1994). A human homologue of the drosophila developmental gene notch is expressed in CD34+ hematopoietic precursors. Blood.

[24] Lampreia FP, Carmelo JG, Anjos-Afonso F (2017). Notch signaling in the regulation of hematopoietic stem cell. Curr. Stem Cell Rep..

[25] Barbosa CM, Bincoletto C, Barros CC, Ferreira AT, Paredes-Gamero EJ (2014). PLCγ2 and PKC are important to myeloid lineage commitment triggered by M-SCF and G-CSF. J. Cell Biochem..

[26] Nogueira-Pedro A (2014). Nitric oxide-induced murine hematopoietic stem cell fate involves multiple signaling proteins, gene expression, and redox modulation. Stem Cells.

[27] Dexter TM, Wright EG, Krizsa F, Lajtha LG (1977). Regulation of haemopoietic stem cell proliferation in long term bone marrow cultures. Biomedicine.

[28] Chtanova T (2008). Dynamics of neutrophil migration in lymph nodes during infection. Immunity.

[29] Yang CW, Strong BS, Miller MJ, Unanue ER (2010). Neutrophils influence the level of antigen presentation during the immune response to protein antigens in adjuvants. J. Immunol..

[30] Brackett CM, Muhitch JB, Evans SS, Gollnick SO (2013). IL-17 promotes neutrophil entry into tumor-draining lymph nodes following induction of sterile inflammation. J. Immunol..

[31] Castell, S. D., Harman, M. F., Morón, G., Maletto, B. A. & Pistoresi-Palencia, M. C. Neutrophils which migrate to lymph nodes modulate CD4(+) T cell response by a PD-L1 dependent mechanism. *Front. Immunol*. **29**, 105 (2019). 10.3389/fimmu.2019.00105 PMC6362305 30761151

[32] Parsa R (2016). BAFF-secreting neutrophils drive plasma cell responses during emergency granulopoiesis. J. Exp. Med..

[33] Hägglöf T (2016). Neutrophils license iNKT cells to regulate self-reactive mouse B cell responses. Nat. Immunol..

[34] Costa S, Bevilacqua D, Cassatella MA, Scapini P (2019). Recent advances on the crosstalk between neutrophils and B or T lymphocytes. Immunology.

[35] Puga I (2011). B cell-helper neutrophils stimulate the diversification and production of immunoglobulin in the marginal zone of the spleen. Nat. Immunol..

[36] Cerutti A, Puga I, Magri G (2013). The B cell helper side of neutrophils. J. Leukoc. Biol..

[37] Woodfin A (2011). The junctional adhesion molecule JAM-C regulates polarized transendothelial migration of neutrophils in vivo. Nat. Immunol..

[38] Starnes TW, Huttenlocher A (2012). Neutrophil reverse migration becomes transparent with zebrafish. Adv. Hematol..

[39] Yang P, Li Y, Xie Y, Liu Y (2019). Different faces for different places: heterogeneity of neutrophil phenotype and function. J. Immunol. Res..

[40] Puhl, S. L. & Steffens, S. Neutrophils in post-myocardial infarction inflammation: damage vs. resolution? *Front. Cardiovasc. Med*. **6**, 25 (2019). 10.3389/fcvm.2019.00025 PMC6431642 30937305

[41] Asada N (2017). Differential cytokine contributions of perivascular haematopoietic stem cell niches. Nat. Cell Biol..

[42] Akashi K, Traver D, Miyamoto T, Weissman IL (2000). A clonogenic common myeloid progenitor that gives rise to all myeloid lineages. Nature.

[43] Kim HS (2018). Activation of formyl peptide receptor 2 by WKYMVm enhances emergency granulopoiesis through phospholipase C activity. BMB Rep..

[44] Maillard I, Adler SH, Pear WS (2003). Notch and the immune system. Immunity.

[45] Duncan AW (2005). Integration of notch and Wnt signaling in hematopoietic stem cell maintenance. Nat. Immunol..

[46] Maderna P (2010). FPR2/ALX receptor expression and internalization are critical for lipoxin A4 and annexin-derived peptide-stimulated phagocytosis. FASEB J..

[47] Pupjalis D, Goetsch J, Kottas DJ, Gerke V, Rescher U (2011). Annexin A1 released from apoptotic cells acts through formyl peptide receptors to dampen inflammatory monocyte activation via JAK/STAT/SOCS signaling. EMBO Mol. Med..

[48] Cooray SN (2013). Ligand-specific conformational change of the G-protein-coupled receptor ALX/FPR2 determines proresolving functional responses. Proc. Natl. Acad. Sci. USA.

[49] Kitsos CM (2005). Calmodulin-dependent protein kinase IV regulates hematopoietic stem cell maintenance. J. Biol. Chem..

[50] Barbosa CM (2011). Differentiation of hematopoietic stem cell and myeloid populations by ATP is modulated by cytokines. Cell Death Dis..

[51] Pantaleão L (2018). Connections of annexin A1 and translocator protein-18 kDa on toll like receptor stimulated BV-2 cells. Exp. Cell Res..

[52] De Buck M (2018). COOH-terminal SAA1 peptides fail to induce chemokines but synergize with CXCL8 and CCL3 to recruit leukocytes via FPR2. Blood.

[53] Paredes-Gamero EJ, Leon CM, Borojevic R, Oshiro ME, Ferreira AT (2008). Changes in intracellular Ca2+ levels induced by cytokines and P2 agonists differentially modulate proliferation or commitment with macrophage differentiation in murine hematopoietic cells. J. Biol. Chem..

