# Supplementary figures and images for: Extracellular annexin-A1 promotes myeloid/granulocytic differentiation of hematopoietic stem/progenitor cells via the Ca2+/MAPK signalling transduction pathway
Source: Cell Death Discov. 2019 Sep 23;5:135. doi: 10.1038/s41420-019-0215-1 (PMC6755131; doi:10.1038/s41420-019-0215-1)

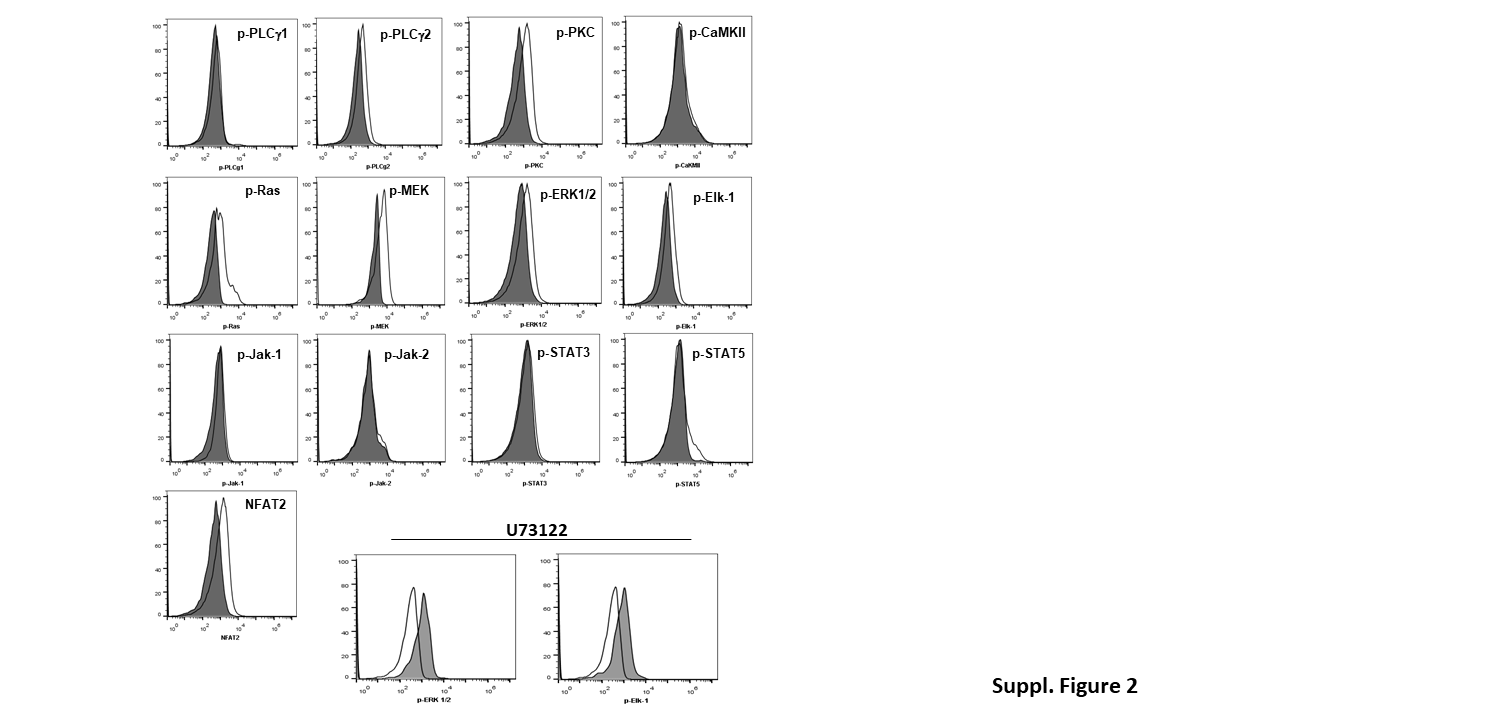

Supplement: Supplementary file 1 — Suppl. Figure 2 [file 41420_2019_215_MOESM1_ESM.tif]

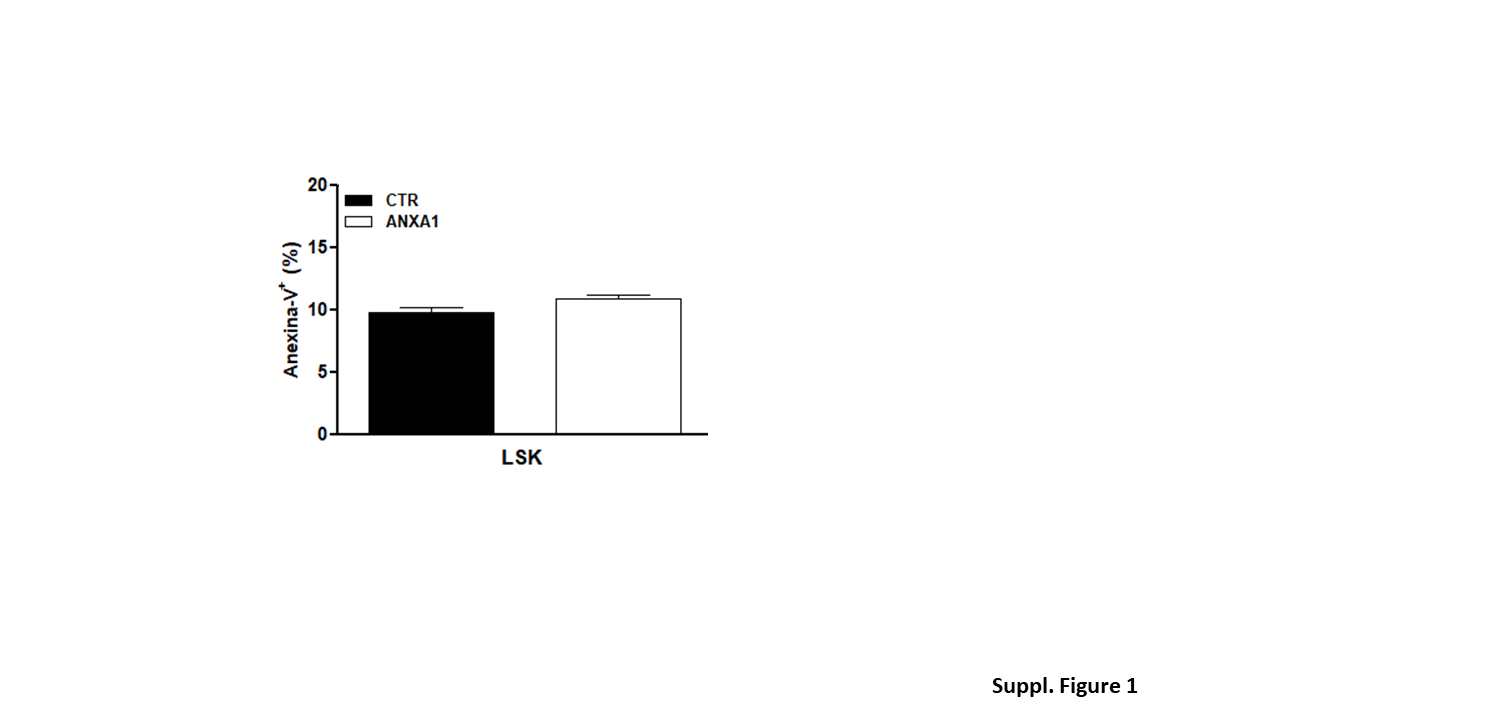

Supplement: Supplementary file 2 — Suppl. Figure 1 [file 41420_2019_215_MOESM2_ESM.tif]
