# Supplementary material for: Extracellular annexin-A1 promotes myeloid/granulocytic differentiation of hematopoietic stem/progenitor cells via the Ca2+/MAPK signalling transduction pathway
Source: Cell Death Discov. 2019 Sep 23;5:135. doi: 10.1038/s41420-019-0215-1 (PMC6755131; doi:10.1038/s41420-019-0215-1)
Supplement: Supplementary file 3 — Supplemental Material File #1 [file 41420_2019_215_MOESM3_ESM.docx]

**Supplementary files**

**Figure 1S:** Effects of rAnxA1 on LSK cell viability. Results referring to viability in Control (n=5) and rAnxA1 (n=5) LSK cells are expressed as mean ± SEM. n=represents the number of animals evaluated.

**Figure 2S:** Representative histograms of the expressions of p-PLCγ1_Tyr783_, p-PLCγ2_Tyr759_, p-CaMKII_thr286_, p-PKC_Thr514_, p-JAK-1_Tyr1022/1023_, p-JAK-2_Tyr1008_, p-Ras, P-MEK, p-ERK1/2_Thr202/Thr204_, p-STAT3_Tyr705_, p-STAT5_Tyr694_, p-Elk-1, NFAT1 and NFAT2 in LSK cells treated with rAnxA1. Representative histograms of the expression of p-ERK and p-Elk-1 in LSK cells treated with PLC inhibitor.
